# The impact of abutment manufacturing techniques on reverse torque after cyclic loading and implant-abutment interface fitting: an in vitro study

**DOI:** 10.1186/s12903-026-09530-w

**Published:** 2026-08-20

**Authors:** Alaa Mostafa AboRas, Ahmed Ismail Taha, Nourhan Ahmed Ragheb

**Affiliations:** 1 Prosthodontic Department, Faculty of Dentistry, Kafr Al Sheikh University, Mubark Road, Kafr Al Sheikh, 33511 Egypt; 2 Kafr Abu Tabl, Kafrelsheikh Governorate, 6860404 Egypt

**Keywords:** Implant, Abutment, Removal torque, Screw loosening, CAD-CAM, Selective laser melting

## Abstract

**Background:**

Screw loosening and implant-abutment interface (IAI) are critical for the long-term success of implant-supported restorations. This study aimed to evaluate the effect of abutment manufacturing technique on reverse torque values and micro-gap measurements at the implant-abutment interface.

**Methods:**

Thirty dental implant-abutment-crown complexes (Implance, Verdent, Turkey) were randomly allocated into three groups (*n* = 10 each) based on the abutment manufacturing technique: Group I—prefabricated titanium abutments (manufacturer-supplied, Implance, Verdent, Turkey; titanium grade 5**)**; Group II—CAD-CAM-milled titanium abutments; Group III—3D-printed titanium abutments (Selective Laser Melting, SLM). All abutments featured an internal hexagonal connection with platform switching. All crown abutments were tightened to 25 Ncm and subjected to cyclic loading (100 N, 1.6 Hz, 250,000 cycles). Removal torque was measured before and after loading. Micro-gaps at the implant-abutment and abutment-screw interfaces were evaluated using scanning electron microscopy (SEM). Statistical analysis was performed using One-Way ANOVA with Tukey’s Post Hoc test for reverse torque and the Kruskal-Wallis test for gap measurements (α = 0.05).

**Results:**

After cyclic loading, the 3D-printed (SLM) abutments exhibited significantly higher reverse torque values (11.08 ± 0.11 Ncm) compared to CAD-CAM-milled abutments (6.40 ± 0.79 Ncm) (*P* < 0.001). Prefabricated abutments demonstrated the highest post-loading torque values (13.31 ± 0.31 Ncm). All intragroup comparisons showed statistically significant reductions from baseline (*P* < 0.0001). No significant difference was found in implant-abutment interface gaps among groups (*P* = 0.105).

**Conclusion:**

Within the limitations of this study, prefabricated abutments demonstrated the highest reverse torque values after cyclic loading. 3D-printed abutments showed significantly greater resistance to torque loss compared to CAD-CAM-milled abutments, approaching the performance of prefabricated abutments. The implant-abutment fit was comparable across groups, suggesting that connection design may be more influential than manufacturing method for macroscopic fit.

3D-printed titanium abutments demonstrated significantly greater mechanical stability than CAD-CAM-milled abutments, approaching the performance of the gold-standard prefabricated abutments. This suggests that additive manufacturing may offer a clinically viable alternative to milling when both customization and mechanical integrity are required. However, clinical studies are needed to confirm these in vitro findings.

## Background

Dental implants have become a highly reliable and widely utilized treatment modality for the rehabilitation of partially edentulous patients, offering predictable long-term outcomes. Systematic reviews and meta-analyses of clinical studies report 5-year survival rates of 94–97% and 10-year survival rates of 89–95% for implant-supported single crowns [1, 2]. Nevertheless, despite these favorable success rates, biological and mechanical complications remain a clinical concern.

The implant-abutment interface (IAI) represents a critical junction in implant-supported restorations. When an abutment is seated onto the implant body and secured with a screw, a degree of misfit is inherently present between the abutment and the internal interface of the implant [3]. This misfit may lead to mechanical complications such as screw loosening or fracture, as well as biological issues including bacterial infiltration, crestal bone loss, and potential loss of osseointegration [4]. Among these, abutment screw loosening is recognized as the most prevalent prosthetic complication [5]. It results from factors such as excessive functional or parafunctional forces, non-passive seating, inadequate or excessive screw torque, and occlusal design [6, 7].

Screw loosening is a two-stage process: initially, external forces induce micromovement and reduce screw preload—the tensile force that secures the screw threads and clamps the components together. Once preload falls below a critical threshold, external forces cause the screw threads to rotate, resulting in loosening [8]. Reverse torque value (RTV) measurement is widely used to assess the force required to initiate screw loosening and serves as an indirect indicator of screw joint stability [9, 10].

The stability of the screw joint is heavily influenced by the precision and design of the abutment-implant interface, making the choice of abutment manufacturing method clinically significant [11]. Prior to the advent of CAD-CAM technology, clinicians relied primarily on prefabricated (stock) abutments or laboratory-cast custom abutments. While prefabricated abutments offer a predictable fit, they may present aesthetic limitations and a reduced ability to customize emergence profiles [12].

The introduction of CAD-CAM systems has enabled the production of custom abutments through either subtractive (milling) or additive (3D printing) manufacturing. Unlike prefabricated abutments with fixed geometries, CAD-CAM allows for individualized design of the emergence profile and subgingival contours, enabling the clinician to achieve superior soft-tissue support and a more natural cervical transition, thereby improving aesthetics, particularly in cases with thin gingival biotypes or high smile lines [13, 14].

Additive manufacturing, particularly selective laser melting (SLM), offers advantages such as material efficiency, reduced production time, and the ability to fabricate complex geometries without tool wear [15, 16]. Titanium remains the material of choice for abutments due to its biocompatibility, corrosion resistance, and favorable mechanical properties [16, 17].

Studies comparing the mechanical performance of different abutment manufacturing methods have yielded conflicting results. While some authors report superior stability with prefabricated abutments [6, 18], others suggest that CAD-CAM fabricated abutments can achieve comparable or even superior mechanical properties when properly designed [19, 20]. These conflicting findings create uncertainty for clinicians selecting abutments, underscoring the need for direct comparative studies under standardized conditions. However, it should be noted that the rougher surface topography of as-built 3D-printed abutments may also present disadvantages, including increased bacterial adhesion and potential stress concentration zones that could predispose to fatigue failure [16].

The accuracy of fit at the IAI is essential for minimizing micromovement, reducing stress transmission to peri-implant bone, and preserving marginal bone levels [4]. While various methods exist to evaluate micro-gaps at the IAI—including radiography, optical microscopy, and microtomography—scanning electron microscopy (SEM) is a well-documented and reliable technique for such assessments [21].

While individual studies have compared prefabricated versus CAD-CAM abutments [6, 19], or milled versus printed abutments [20], no study has directly compared all three manufacturing techniques using identical implant connection geometry, cyclic loading protocols, and comprehensive SEM analysis of both interfaces. This direct three-way comparison is essential for evidence-based clinical decision-making regarding abutment selection. To our knowledge, this is the first study to directly compare all three manufacturing techniques (prefabricated, milled, and 3D-printed) using identical implant connection geometry, standardized crown design, and comprehensive SEM analysis of both interfaces.

Despite the growing use of custom abutments, direct comparative data on the mechanical performance of 3D-printed versus CAD-CAM-milled abutments under cyclic loading remains scarce [3, 19]. The specific aims of this in vitro study were twofold: (1) To evaluate the effect of the abutment manufacturing technique (prefabricated, CAD-CAM-milled, and 3D-printed selective laser melting) on the reverse torque values of abutment screws after cyclic loading, as a measure of screw joint stability and mechanical performance. (2) To measure and compare the micro-gaps at the implant-abutment interface and abutment-screw interface for the three abutment types using scanning electron microscopy, as an indicator of interface fit quality.

The proposed null hypotheses were that: (1) There is no significant difference in the reverse torque values of abutment screws among prefabricated, CAD-CAM-milled, and 3D-printed abutments after cyclic loading. (2) There is no significant difference in the micro-gap at the implant-abutment interface among the three abutment types.

## Materials and methods

This in vitro study was approved by ethical committee of the Faculty of Oral and Dental Medicine and Surgery, Kafrelsheikh University, Kafr El-Sheikh, Egypt under number (KFSIRB200-370).

### Sample size calculation

Sample size calculated depending on a previous study [22]. According to this study, when mean ± standard deviation of torque loss in Group I is 20.12 ± 3.36, while estimated difference was 4.5 [23], when the (power) is 0.80 and the Type I error probability associated with this test is 0.05. The minimum sample size needed is 10 per group. The sample size was calculated using T test which was performed by using P.S.Power 3.1.2.

Thirty implants (Implance, Verdent, Turkey; Ø4.8 mm, length 14 mm, internal hexagonal connection with platform switching) were randomly allocated into three experimental groups: Group I: Prefabricated titanium abutments (manufacturer-supplied, Implance, Verdent, Turkey; titanium Grade 5; internal hexagonal connection; lot number :25072904000); Group II: CAD-CAM-milled titanium abutments (Coritec 250i, imes-icore GmbH; from solid titanium blocks, Grade 4 titanium); Group III: 3D-printed titanium abutments (SLM, DUAL-150, Riton, Japan) using Ti-6Al-4 V powder (grain size 15–45 μm according to ASTM F136 standard).

### Mold preparation

A typodont model (Nissan Cast) was used to simulate the mandibular arch; the lower right six was removed, and an implant was installed in the socket by using pink wax. A dental surveyor (Ney Surveyor) was used to ensure accurate three-dimensional alignment, optimal angulation, and consistent fixture positioning at the simulated alveolar bone level. An impression was made with addition-silicone material (Elite HD+, Zhermack, Italy**)** for its high dimensional stability, followed by pouring an epoxy resin model (EpoxiCure 2, Buehler, USA**)**. Each implant-abutment assembly was embedded in an epoxy resin 2 ml below the gingival crest. Resin was poured incrementally over a vibrator to minimize polymerization shrinkage.

To create a clinically representative emergence profile for digital scanning, a temporary abutment supplied by the manufacturer was seated in the position of the lower first molar (tooth #46) within the gingival former, simulating intraoral conditions, including the emergence profile and peri-implant soft tissue contours, during specimen preparation. Subsequently, a temporary crown resin material (Protemp4, 3 M ESPE, Germany) was injected directly over the abutment to capture the emergence profile by adapting the material around the abutment to replicate the natural tooth contour, creating a customized emergence profile shape that mimics the natural emergence angle and soft tissue support and allowing it to set according to the manufacturer’s instructions before removal and finishing. The abutment with temporary material was secured to the implant in the model to represent the oral condition and to make the gingival contour using gingival mask material (Xilgum, LASCOD, Italy). A gingival mask material was used to replicate peri-implant soft tissue contours.

### Fabrication of customized titanium abutments

**Fig. 1:**
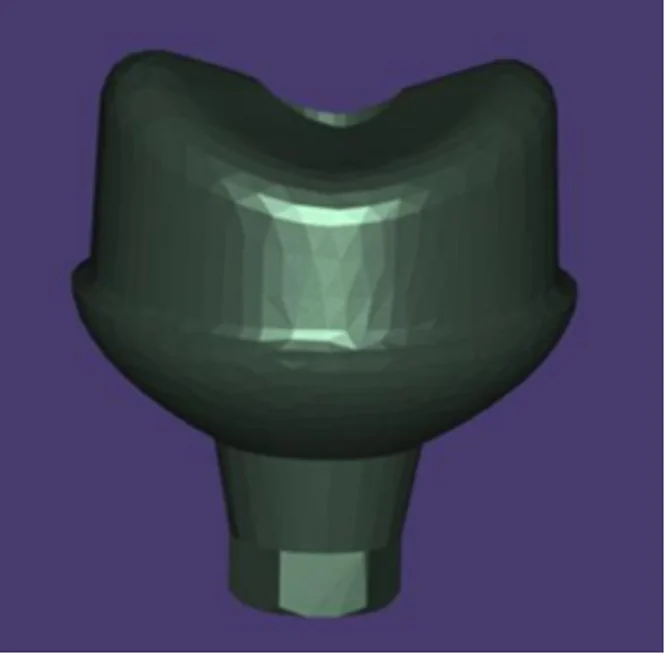
Virtual design of the customized abutment showing the implant connection interface and coronal morphology

After implant installation in the epoxy blocks and gingival form creation, a scan body (PSB, a bone-level scan body) supplied by the manufacturer was screwed into the implant and scanned using an intraoral scanner (Medit i700, MEDIT Corp., Seoul, Republic of Korea) to detect its position and the connection of the implant; then the scan was exported to the CAD software (Exocad, Darmstadt, Germany). The CAD workflow proceeded as follows: (1) The scan body file was imported into Exocad software to establish the implant position and orientation. (2) The abutment platform was selected from the manufacturer’s digital library, as shown in Fig. 1. (3) The custom abutment was designed using the implant position data derived from the scan body file to ensure accurate positioning. (4) The same STL file was used for both milling and printing to ensure identical geometric specifications.

**Fig. 2:**
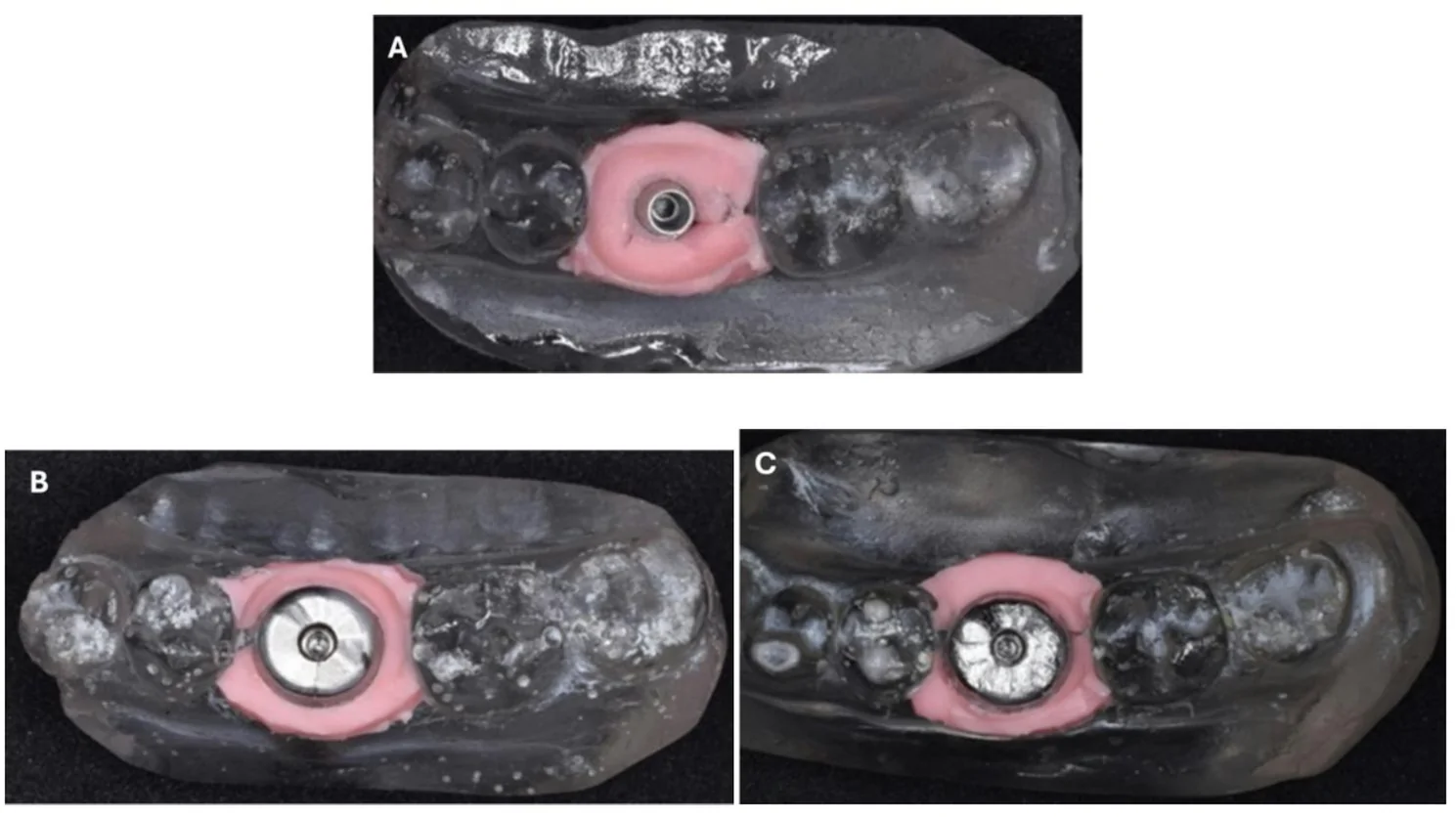
Representative specimens from each group: **A** prefabricated abutment with a crown, **B** CAD-CAM milled abutment with a crown, and (**C**) 3D-printed abutment with a crown. All crowns were designed with standard mandibular first molar anatomy with screw access holes

The STL files obtained from the digital scans were used to design two types of customized titanium abutments (milled and printed) (*n* = 10). Milled abutments are produced from solid titanium blocks using a CAD-CAM subtractive 5-axis milling machine (Coritec 250i, imes-icore GmbH, Eiterfeld, Germany), and 3D-printed abutments are manufactured via metal additive manufacturing (selective laser melting) with a layer-by-layer construction process using Ti 6Al-4 V powder with a grain size of 15–45 μm using (DUAL-150, Riton, Japan). Both milled and 3D-printed abutments underwent no additional surface polishing or post-processing after fabrication, except for ultrasonic cleaning in isopropyl alcohol. This decision was made to isolate the manufacturing technique as the primary variable; however, we acknowledge that clinical practice often includes surface finishing procedures. Both abutment types were fabricated using the same virtual design parameters to ensure comparability, as shown in (Fig. 2A-C).

### Crown fabrication for prefabricated abutments (Group I)

**Fig. 3:**
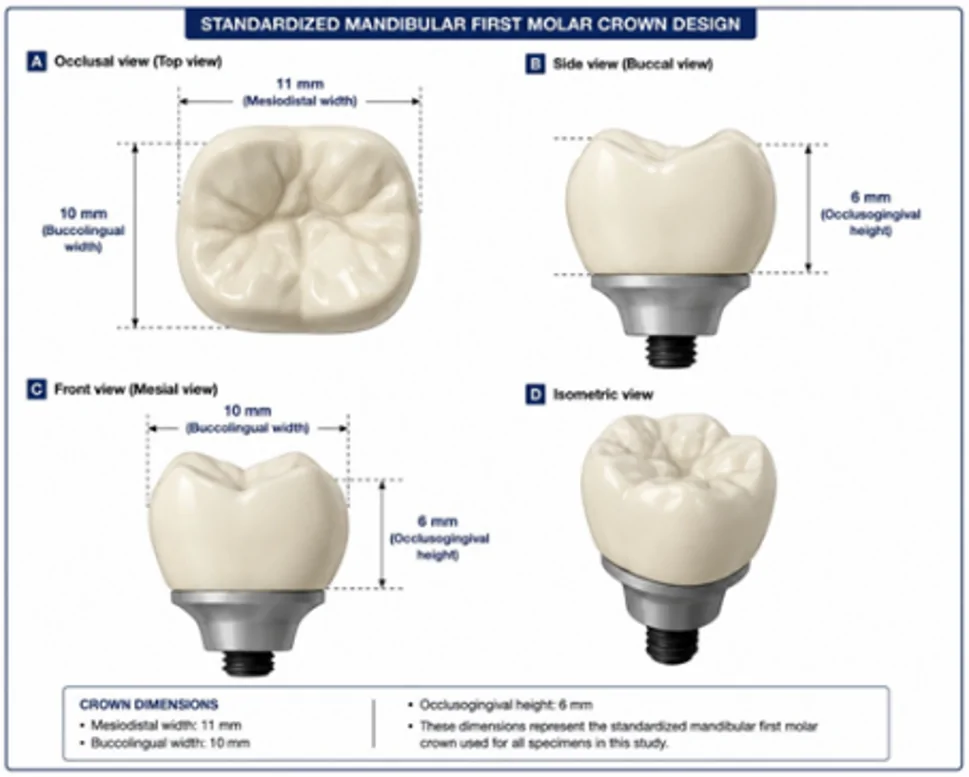
Schematic figure of the crown was designed using standard mandibular first molar dimensions

For prefabricated abutments, the crown fabrication workflow followed a different procedure compared to customized abutments. The prefabricated abutment was first scanned using an intraoral scanner (Medit i700, MEDIT Corp., Seoul, Republic of Korea) to capture its exact geometry. The scanned abutment file was imported into Exocad software as a reference object. The crown was then designed directly over the prefabricated abutment’s digital model using the same anatomical parameters (mesiodistal: 11 mm, buccolingual: 10 mm, occlusogingival: 6 mm), margin configuration (chamfer finish line, 0.5 mm width), and cement space (50 μm) as used for customized abutments, as shown in Fig. 3. This ensured that all crowns had identical external morphology, regardless of the underlying abutment type. The screw access hole was positioned in the central fossa to allow for prosthesis retrievability.

### Crown fabrication for customized abutments

(Groups II and III): For CAD-CAM-milled and 3D-printed abutments, the crown was designed simultaneously with the abutment using the same CAD software. The crown geometry was designed to precisely fit the customized abutment’s preparation, using the same anatomical parameters, margin configuration, and cement space (50 μm) to maintain standardization across all groups. The crown was designed with a screw access hole positioned in the central fossa. The crown and abutment were designed as a virtual assembly to ensure accurate fit but were fabricated as separate components. The crown design was exported as a separate STL file for milling.

### Fabrication of final restorations

Thirty monolithic zirconia crowns (Ceramill zolid ht+ white; Amann Girrbach AG, Koblach, Austria) were designed in the lower first molar anatomy with screw access holes using CAD software (Exocad, Darmstadt, Germany). The zirconia crowns were milled using a milling unit (Imes-Icore, Germany) using 5-axis machining with standard burs. After milling, the crowns were sintered using a sintering furnace at 1530 °C with a dwell time of 2 h according to the manufacturer’s recommendations, then finished and polished using diamond-impregnated silicone polishers to achieve a smooth surface. The crowns were then cemented to abutments using dual-cure resin cement (PANAVIA™ V5, Kuraray Europe GmbH, Germany). Crowns were checked for complete seating using a dental explorer evaluation before cementation. Surface treatment before cementation: Titanium abutment surfaces were airborne-particle abraded with 50 μm alumina particles at 0.25 MPa pressure from a standardized distance of 30 mm at a 90° angle for 10 s [24]. Zirconia crown intaglio surfaces were sandblasted with 50 μm alumina at 0.2 MPa from a standardized distance of 10 mm at a 90° angle for 10 s, ultrasonically cleaned in isopropanol for 5 min, and treated with 10-MDP-containing primer (Clearfil Ceramic Primer, Kuraray) applied with a microbrush and air-dried [25]. This standardized surface treatment protocol was applied identically to all crown-abutment combinations to eliminate surface-related variables. Then, all abutments and restorations were ultrasonically cleaned with isopropyl alcohol for 3 min and then dried. Dual-cure resin cement was applied and light-cured for 20 s per surface using an LED curing light (Elipar S10, 3 M ESPE) with an irradiance of ≥ 1000 mW/cm² to the restoration according to the manufacturer’s recommendations, and excess cement was removed. Excess cement was carefully removed using a dental explorer and floss before complete polymerization to prevent cement-related complications. Following cementation, all crown-abutment complexes were stored in distilled water at 37 °C for 24 h before baseline torque testing to allow for complete cement polymerization and stabilization. This standardized crown fabrication and cementation protocol ensured that any observed differences in reverse torque values could be attributed to the abutment manufacturing technique rather than variations in crown design, fit, or cementation.

### Initial torque test (baseline measurement)

**Fig. 4:**
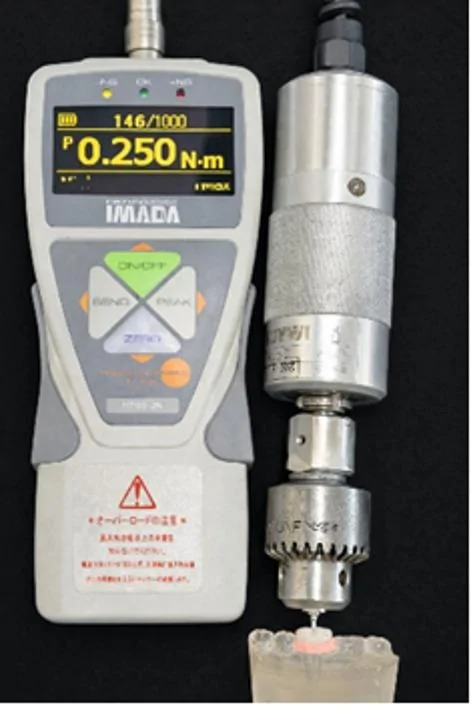
Digital torque device after crown-abutment complex was seated and tightened to the manufacturer’s recommended load (25 Ncm)

For baseline measurement, each crown-abutment complex was seated and tightened to the manufacturer’s recommended load (25 Ncm) using a digital torque gauge (HTG2–200Nc, IMADA, Toyohashi, Japan) as shown in Fig. 4. After an initial 10-minute period to allow for settling, all screws were retightened to 25 Ncm. Following a second 10-minute interval, the reverse torque value (RTV) required to initiate screw loosening was measured and recorded as the ‘pre-loading’ baseline, a protocol consistent with prior in vitro studies [26]. The maximum torque needed for initial loosening was recorded in Newton-centimeters (Ncm). These values were used for subsequent comparison with post-loading reverse torque measurements to quantify torque loss after cyclic loading.

### Mechanical cyclic loading test

All specimens underwent a mechanical cyclic loading simulator machine (Robota, Egypt) with a vertical force of 100 N and a frequency of 1.6 Hz at the crown center along the implant’s long axis for 250,000 cycles. This corresponds to approximately one year of clinical function [27, 28], with a stylus rod size of 6 positioned at the center of the crown along the implant’s long axis in a vertical direction. The test conditions were maintained at room temperature and wet conditions (distilled water). Screw loosening was evaluated after the cyclic loading procedure.

### Reverse torque test (post-cyclic loading)

After mechanical cyclic loading, the torque required to unscrew the abutment screws was measured using the same digital torque gauge with the same procedures as measuring initial RTV. These values were statistically compared to determine the influence of abutment manufacturing technique and abutment-screw complex on screw stability under simulated aging conditions.

### Calculation of Removal Torque Loss (RTL) ratio of abutment screw before and after cyclic loading

Screw loosening of each assembly (implant-abutment) was analyzed by measuring RTV before and after dynamic cyclic load by using the digital torque gauge. Removal torque loss (RTL) can be an indicator of how much loosening takes place. This metric has been widely used in in vitro studies to quantify screw joint stability and predict clinical loosening risk [9, 10, 26]. The percentage torque loss was calculated as $\left[\left(\mathrm{pre-loading}-\mathrm{post-loading}\right)/\mathrm{pre-loading}\right]\times100\%$.

### SEM observation abutment-screw complex configuration measurement

Implant-abutment assemblies were embedded in epoxy resin and longitudinally sectioned using (Isomet^®^4000, BUEHLER, Germany) under continuous water cooling. Sectioning was performed slowly with copious coolant to minimize specimen deformation and gap alterations. The slow cutting speed and continuous irrigation prevented heat generation that could cause thermal expansion or distortion of the abutment-screw interface. Additionally, the specimens were firmly secured in the cutting fixture to prevent movement during sectioning. The sectioned surfaces were subsequently ground and polished using a micro-grinding system with silicon carbide papers ranging from 800 to 4000 grit under vacuum conditions to obtain a smooth surface suitable for microscopic evaluation.

The screw-abutment interface was examined using a scanning electron microscope (SEM) under magnifications (x2000) to observe the gap throughout the interface.

**Fig. 5:**
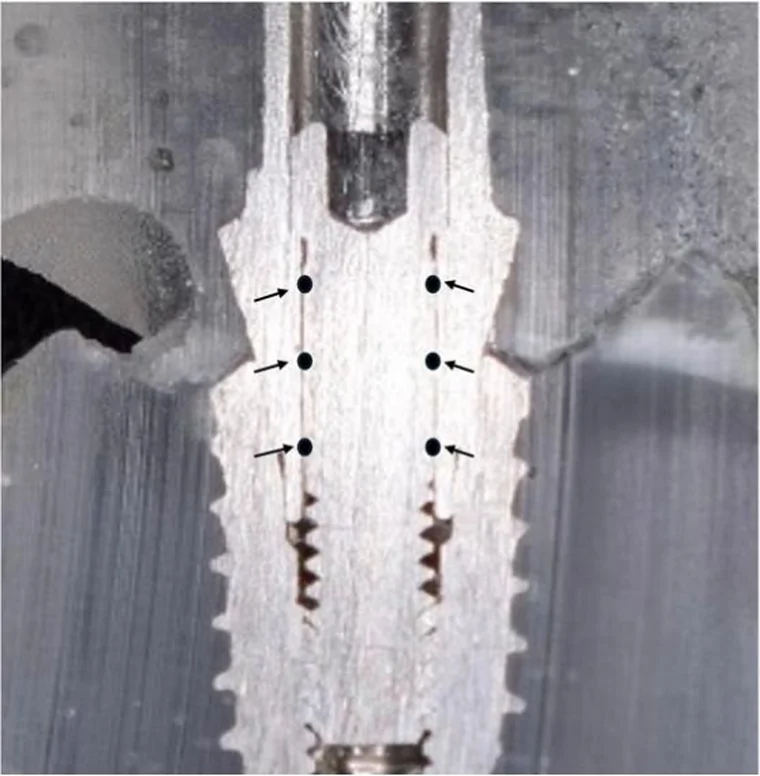
Cross-section of implant abutment screw complex demonstrating the areas for measuring gaps in abutment-screw interface

**Fig. 6:**
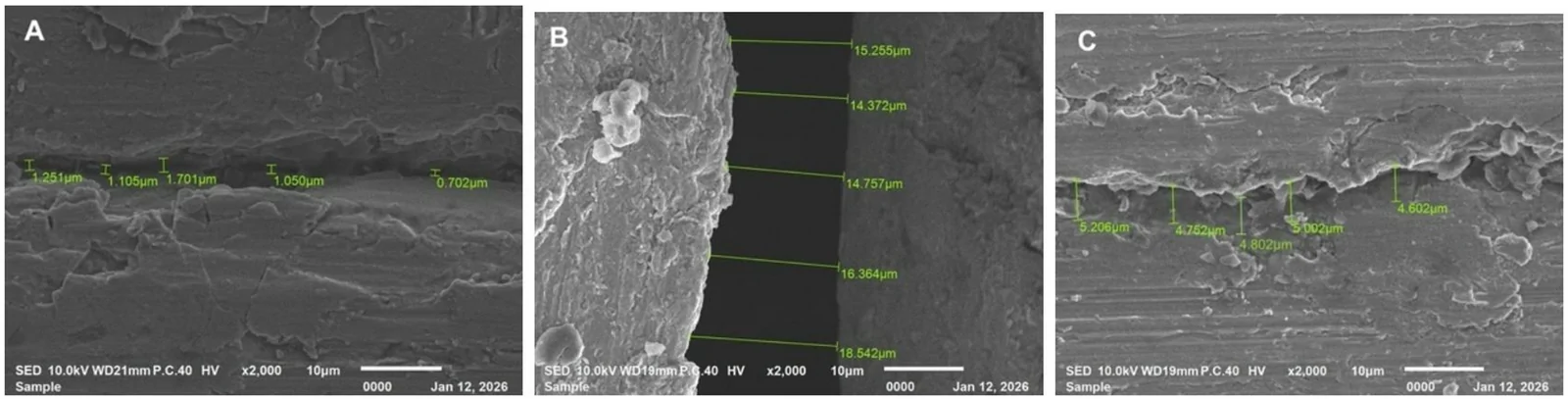
SEM images (2000X) of abutment-screw interface gaps: **A** Prefabricated abutment, **B** CAD-CAM milled abutment, **C** 3D-printed abutment

Multiple readings were taken at three standardized areas along the screw-abutment interface for each specimen: (1) the cervical region near the screw head, (2) the middle region of the screw threads, and (3) the apical region near the screw tip, as shown in Fig. 5. These areas were selected to represent the entire screw-abutment interface from the screw head to the apical tip, capturing potential variations in fit along the screw length. The measurement points were standardized across all specimens using the anatomical landmarks of the screw threads. Measurements were obtained in micrometers using SEM image analysis software as shown (Fig. 6A-C), and mean values were calculated for each specimen and subsequently used for statistical analysis for each group.

### SEM observation of implant-abutment interface

**Fig. 7:**
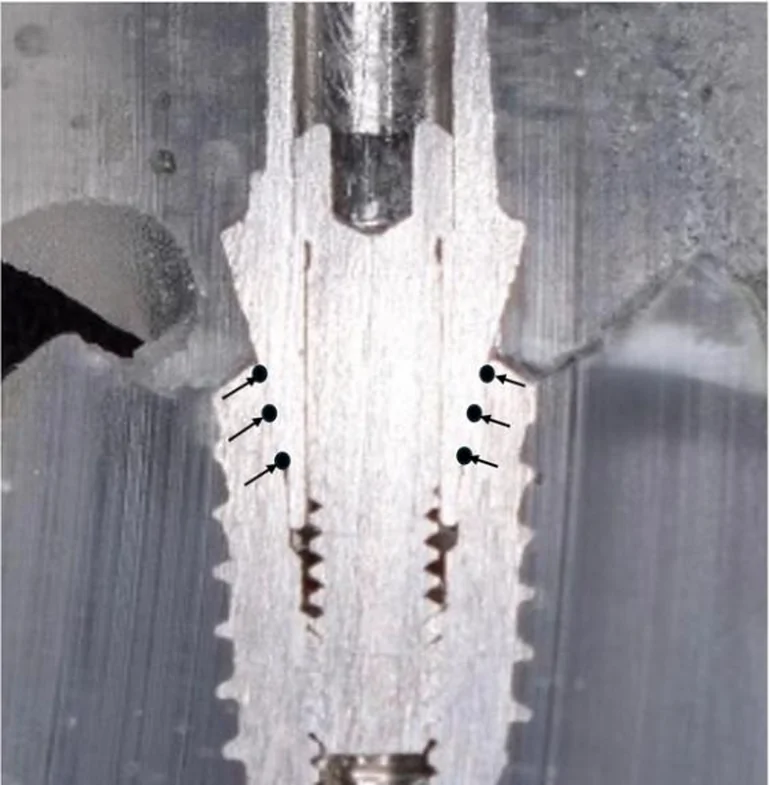
Cross-section showing measurement areas for implant-abutment interface gaps

**Fig. 8:**
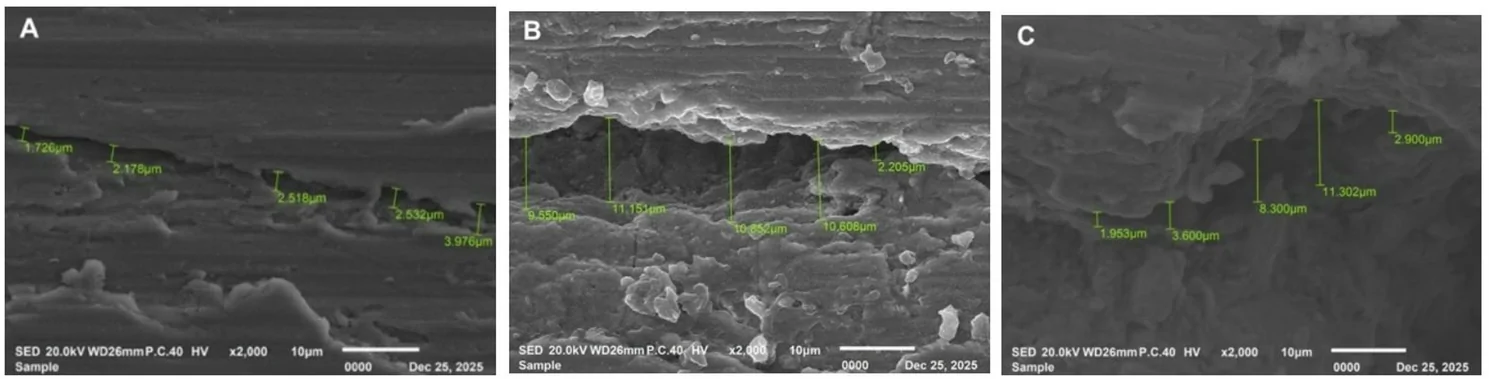
SEM images (2000X) of implant-abutment interface gaps: **A** Prefabricated abutment, **B** CAD-CAM milled abutment, **C** 3D-printed abutment

Following specimen preparation and sectioning, the implant-abutment interface was evaluated using scanning electron microscopy (SEM) to assess the interfacial adaptation between the implant fixture and the abutment. The sectioned specimens were examined under magnifications of 2000X. Six standardized measurement points at the implant-abutment interface were selected for each specimen, including the buccal external point, middle point, internal point, lingual external point, middle point, and internal point, as shown in Fig. 7, and then their micro-gaps were observed using SEM, as shown in Fig. 8A-C.

To avoid the potential for researcher bias, multiple readings were obtained for each specimen, recorded in micrometers, and the mean values were calculated for each specimen. Measurements were performed by two calibrated examiners who were blinded to group allocation. Interobserver reliability for all gap measurements was rigorously assessed using the Intraclass Correlation Coefficient (ICC) with SPSS 27, GraphPad Prism, and Microsoft Excel 2016. The findings demonstrate excellent agreement between the two observers across all experimental groups. The CC values for both abutment-implant and abutment screw gaps ranged from 0.995 to 1.000, indicating near-perfect to perfect reliability for the prefabricated, CAD/CAM milled, and 3D printing groups. The very narrow 95% confidence intervals, all approaching 1.0, further confirm the high precision and consistency of the measurement method. All observed ICC values were statistically significant (*P* < 0.0001), thereby validating the robustness and reproducibility of the assessment technique across all fabrication methods. These values were subsequently used for statistical analysis to compare the implant-abutment fit among the different experimental groups.

### Statistical analysis

The statistical analysis was performed in consultation with a biostatistician using SPSS 27^®^ (IBM Corp., Armonk, NY, USA), Graph Pad Prism^®^ (Version 9.0, GraphPad Software, San Diego, CA, USA), and Microsoft Excel 2016. Normality was assessed using Shapiro-Wilk and Kolmogorov-Smirnov tests. Reverse torque data (normally distributed) were analyzed using One-Way ANOVA with Tukey’s HSD post hoc test for pairwise comparisons. Gap measurements (non-normally distributed as determined by the Shapiro-Wilk test, *p* < 0.05) were analyzed using the Kruskal-Wallis non-parametric test with Dunn’s post hoc test with Bonferroni correction for multiple comparisons. Paired t-tests were used for intragroup comparisons of torque before and after loading. Significance was set at α = 0.05.

## Results

### Measuring reverse torque

The mean post-loading reverse torque values were 13.31 ± 0.31 Ncm (95% CI: 13.08–13.54) for prefabricated abutments, 6.40 ± 0.79 Ncm (95% CI: 5.78–7.02) for CAD-CAM-milled abutments, and 11.08 ± 0.11 Ncm (95% CI: 10.99–11.17) for 3D-printed abutments. Table 1 demonstrated post-loading reverse torque was highest in the prefabricated group (13.31 ± 0.31 Ncm), followed by the 3D-printed group (11.08 ± 0.11 Ncm), and lowest in the CAD-CAM-milled group (6.40 ± 0.79 Ncm, *p* < 0.0001). Torque loss was greatest in the milled group (− 67.99%).

**Table 1:** Comparison between groups regarding reverse torque before and after loading and percentage of change

	Group						*P*-value
	Prefabricated		CAD-CAM milled		3D-Printing		
	Mean	Standard Deviation	Mean	Standard Deviation	Mean	Standard Deviation	
Before loading	19.52	0.31	20.01	0.13	20.64	0.09	< 0.0001*
After loading	13.31	0.31	6.40	0.79	11.08	0.11	< 0.0001*
*P*-value	< 0.0001*		< 0.0001*		< 0.0001*		
% of change	-31.78	1.64	-67.99	4.04	-46.31	0.70	< 0.0001*

*Significant difference as *P* < 0.05

**Fig. 9:**
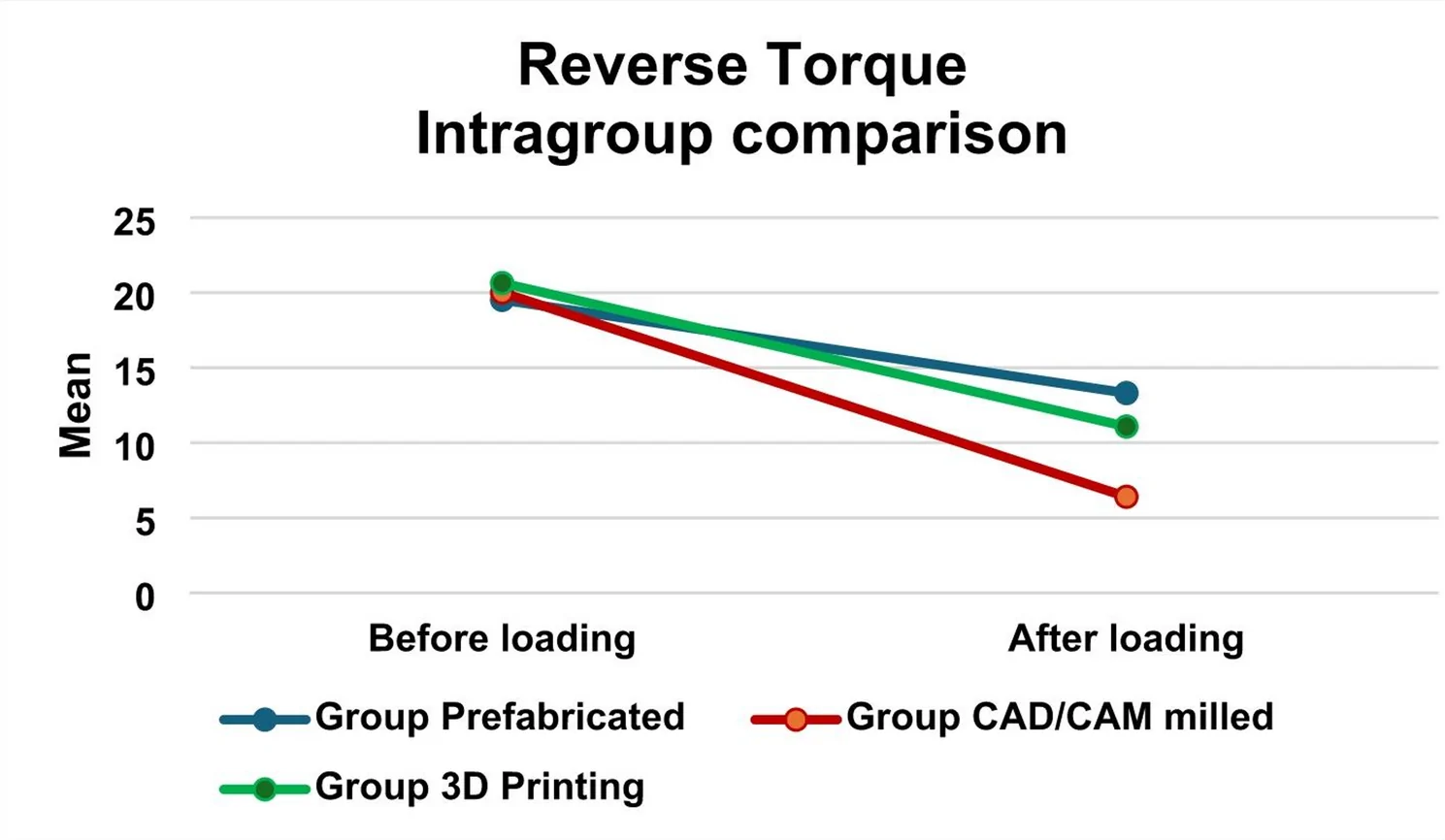
Line chart showing intragroup comparison of reverse torque values before and after cyclic loading. All groups showed statistically significant reductions (*P* < 0.0001, paired t-test)

Intragroup analysis revealed a statistically significant reduction in reverse torque values after loading within each group (*P* < 0.0001).The prefabricated group showed a decrease from 19.52 ± 0.31 before loading to 13.31 ± 0.31 after loading. Similarly, the CAD-CAM milled group demonstrated a marked reduction from 20.01 ± 0.13 to 6.40 ± 0.79, while the 3D-printing group decreased from 20.64 ± 0.09 to 11.08 ± 0.11, as shown in Fig. 9.

### Abutment screw gaps

As presented in Table 2, a statistically significant difference was found among the groups for abutment-screw interface gaps (*p* = 0.01) (Table 2). The CAD-CAM milled group exhibited significantly larger gaps (median: 17.45 μm) compared to both the prefabricated (median: 4.25 μm) and 3D-printed (median: 5.31 μm) groups (*p* < 0.001).

**Table 2:** Comparison between groups regarding abutment-screw gaps using the Kruskal-Wallis test

Group	Minimum	Maximum	Median	Mean	Standard Deviation	*P*-value
Prefabricated	0.70	8.85	4.25 ^**a**^	4.43	3.50	0.01*
CAD-CAM milled	14.37	21.76	17.45 ^**b**^	17.74	2.76	
3D-Printing	3.30	10.35	5.31 ^**a**^	5.94	2.57	

*Significant difference as *P* < 0.05

Medians with different superscript letters are significantly different (*p* < 0.05)

Pairwise comparisons using Dunn’s post-hoc test (Table 3) confirmed these findings. A significant difference was observed between the prefabricated and CAD-CAM milled groups (adjusted *P* = 0.0001), as well as between the 3D-printing and CAD-CAM milled groups (adjusted *P* = 0.0012). However, no statistically significant difference was detected between the prefabricated and 3D-printing groups (adjusted *P* > 0.9999).

**Table 3:** Pairwise comparisons between groups regarding abutment-screw gaps using Dunns test

Sample 1-Sample 2	Mean rank 1	Mean rank 2	Mean rank diff.	Adjusted *P*-value
Prefabricated vs. CAD-CAM milled	9.4	25.5	-16.1	0.0001*
Prefabricated vs. 3D-printing	9.4	11.6	-2.2	> 0.9999
3D-Printing vs. CAD-CAM milled	25.5	11.6	13.9	0.0012*

*Significant difference as *P* < 0.05

### Abutment-implant gaps

No significant difference was found among groups (*p* = 0.105), as shown in Table 4. The prefabricated group showed the smallest median gap (4.24 μm). It is noteworthy that prefabricated abutments demonstrated the smallest median gaps at both interfaces (screw gaps: 4.25 μm; implant-abutment gaps: 4.24 μm), suggesting superior fit quality compared to both customized groups, although the implant-abutment differences did not reach statistical significance.

**Table 4:** Comparison between groups regarding abutment-implant gaps using the Kruskal-Wallis test

Group	Minimum	Maximum	Median	Mean	Standard Deviation	*P*-value
Prefabricated	2.18	5.06	4.24 ^**a**^	3.83	1.10	0.105
CAD-CAM milled	2.00	11.15	6.50 ^**a**^	6.72	3.66	
3D Printing	1.95	15.97	7.45 ^**a**^	7.76	4.42	

Medians with different superscript letters are significantly different (*p*<0.05)

## Discussion

This in vitro study evaluated how abutment manufacturing techniques affect screw joint stability and interface adaptation. The key findings lead to the rejection of the first null hypothesis, as significant differences in reverse torque values were observed after cyclic loading. Conversely, the findings support the acceptance of the second null hypothesis, as no significant difference in implant-abutment micro-gaps was found.

The CAD-CAM-milled abutments exhibited the greatest torque loss (-67.99%) and the largest screw-abutment gaps. This can be attributed to the subtractive manufacturing process, where tool wear and vibration may introduce micro-inaccuracies in the internal screw chamber, reducing the effective contact area for frictional resistance. While tool wear may not directly affect the external abutment surface, which is machined with larger-diameter tools and more accessible geometry, the internal screw chamber—which is critical for screw engagement—is particularly susceptible to machining artifacts due to its complex geometry, narrow access, and limited visibility during milling. This creates a situation where the precision-critical internal surface may have greater variability than the external surface. This likely facilitated micro-sliding and accelerated preload dissipation under cyclic loads [29, 30]. Similar findings were reported by Yi et al. [30], who noted that milled abutments showed greater variance and lower stability in screw joint mechanics compared to conventionally manufactured components, potentially due to machining artifacts and stress concentrations at the screw interface.

The superior torque retention of 3D-printed abutments relative to milled ones may be partly attributed to the potentially rougher as-built surface topography of additive manufacturing, which could enhance frictional resistance at the screw-abutment interface [26]. Conversely, if these abutments underwent post-processing finishing (e.g., polishing), the frictional benefit might be reduced, potentially narrowing the performance gap observed between 3D-printed and milled abutments. This trade-off between mechanical advantage and surface finishing is a critical consideration for clinical implementation. This is supported by Albaijan et al. [20], who found that SLM-fabricated abutments exhibited higher surface micro-roughness and maintained greater removal torque values compared to their milled counterparts, suggesting a beneficial effect of the additive manufacturing surface characteristic on screw stability. However, it should be noted that this surface roughness may also have negative implications, including increased bacterial adhesion and potential stress concentration zones that could predispose it to fatigue failure [16, 31].

Since surface roughness was not quantitatively measured in this study, this remains a proposed mechanism requiring future investigation. Furthermore, the SLM process, building the abutment from powder layer by layer, may achieve a more geometrically congruent internal screw chamber, as evidenced by the significantly smaller micro-gaps observed compared to milled abutments, thereby optimizing the screw’s engagement and clamping force distribution and approaching—though not surpassing—the precision of prefabricated abutments. It should be noted that prefabricated abutments still demonstrated the smallest median gaps (4.25 μm) and highest post-loading torque values (13.31 Ncm), indicating that industrial manufacturing processes remain the gold standard for precision.

Campos et al. [16] also highlighted that the layer-by-layer fabrication in additive manufacturing can lead to more homogeneous internal geometries, potentially reducing points of stress concentration and improving mechanical interlocking. However, additive manufacturing also leaves surface imperfections such as pores and partially fused powder particles attached to the surface, which could create stress concentration zones [31]. This is why some manufacturers combine additive and subtractive methods to achieve optimal surface quality.

Our finding that prefabricated abutments showed the least torque loss is consistent with several studies [6, 18], highlighting the precision and optimized material properties of industrially manufactured components. Hamilton et al. [17] similarly concluded that prefabricated abutments generally demonstrate superior fit and mechanical predictability due to controlled industrial manufacturing processes. The superior performance of 3D-printed abutments relative to milling, however, presents a compelling nuance. It suggests that additive manufacturing can emulate the mechanical stability of prefabricated abutments while offering the customization benefits of CAD-CAM. This challenges the blanket assumption that all customized abutments inherently carry a higher risk of screw loosening [32], indicating that the fabrication method within customization is a decisive factor. The consistently smaller gaps observed in prefabricated abutments across both interfaces highlight the precision achievable through industrial manufacturing processes, reinforcing their role as the reference standard for abutment fit.

The standardized crown dimensions used in this study (mesiodistal: 11 mm, buccolingual: 10 mm, occlusogingival: 6 mm) represent average mandibular first molar dimensions. Variations in crown size, particularly larger crowns in clinical situations, could potentially introduce greater micromovements and stress concentrations at the screw interface, as the lever arm effects would be amplified [7, 33]. This underscores the importance of considering restoration size when selecting abutment types, particularly for posterior restorations.

The applied load of 100 N, while at the lower spectrum of physiological occlusal forces, was sufficient to induce measurable and differential torque loss, underscoring the sensitivity of the screw joint to cyclic fatigue. The dramatic loss in the milled group suggests its performance might further degrade under higher, more clinically representative loads, a critical consideration for posterior restorations. Gocmez et al. [19] also utilized cyclic loading protocols and found that custom abutments, particularly milled ones, were more susceptible to torque loss under simulated masticatory forces, aligning with our observations of significant degradation in the CAD-CAM milled group.

The acceptance of the second hypothesis regarding the implant-abutment interface aligns with the premise that connection geometry is a primary determinant of this fit. The internal hexagon design used here likely imposed a greater influence on the macroscopic interface than the subtleties of the fabrication process, a finding supported by prior research emphasizing the role of connection design over manufacturing method alone [34]. However, it is important to acknowledge that external hex connections may be more susceptible to manufacturing variations, as they rely on external surfaces for alignment rather than internal geometry. Future studies should validate these findings across different connection designs, including external hex and conical connections, as the influence of manufacturing method may vary with connection geometry. For instance, Mishra et al. [35] concluded in their systematic review that the type of implant-abutment connection (e.g., internal hex, conical) has a more significant impact on microleakage and interfacial stability than the abutment fabrication method.

The discrepancy between the significant differences in screw-abutment gaps and the non-significant differences in implant-abutment gaps suggests that the manufacturing method may affect the screw interface more profoundly than the macroscopic connection interface. We hypothesize that the primary mechanism for torque loss is not the macroscopic fit at the implant-abutment junction but rather the microscale adaptation of the screw threads to the abutment’s internal chamber. The larger screw-abutment gaps observed in the CAD-CAM-milled group would permit increased micro-movement at the thread interface under cyclic loading, more rapidly dissipating the screw’s preload and leading to greater torque loss. In contrast, the prefabricated and 3D-printed abutments, with their significantly smaller screw-abutment gaps, maintained more intimate thread contact, preserving preload more effectively. This may explain why torque retention differed while macroscopic fit did not. This suggests that for clinicians, the choice between prefabricated, milled, or printed abutments may not compromise the foundational seal at the implant level, provided the same connection system is used.

The clinical implication of these findings is twofold: First, clinicians can consider 3D-printed abutments as a viable alternative to milled abutments when mechanical stability is a primary concern, as they demonstrated significantly better torque retention (46.31% loss vs. 67.99% loss). Second, in cases where maximum mechanical stability is critical—such as in posterior restorations or patients with parafunctional habits—prefabricated abutments remain the gold standard, as they maintained the highest post-loading torque values (13.31 Ncm).

Alternative explanations for the observed differences include potential variations in material properties (Ti-6Al-4 V for printed vs. Grade 5 titanium for prefabricated and Grade 4 titanium for milled), differences in surface topography, and the possibility that the SLM process introduces beneficial residual compressive stresses that enhance fatigue resistance. Without quantitative surface roughness and residual stress measurements, these mechanisms remain speculative. Within the limitations of this in vitro study, the superior torque retention observed in 3D-printed abutments relative to milled ones suggests a potential mechanical advantage. However, direct translation of these in vitro findings to clinical outcomes should be made with caution, as the complex intraoral environment may influence performance in ways not simulated in this study.

Regarding post-processing: While some SLM manufacturers claim that post-processing is not mandatory for stress release in abutments due to the inherent properties of the Ti-6Al-4 V alloy and the controlled cooling rates during printing, this remains a debated topic in the literature. Stress relief annealing at 650–800 °C can reduce residual stresses that may develop during the layer-by-layer construction process, potentially improving fatigue resistance [31]. However, such heat treatment could also affect dimensional accuracy and surface characteristics. Our findings should be interpreted in the context of as-built conditions, and future studies should systematically evaluate the effect of various post-processing protocols—including stress relief annealing, surface polishing, and combined additive-subtractive approaches—on mechanical performance and interface fit. The absence of post-processing may represent a conservative estimate of clinical performance, as post-processing could potentially improve or, in some cases, compromise the fit and mechanical properties [18, 22].

These findings contribute to the evolving understanding that manufacturing method significantly influences mechanical performance of implant components. The ability of 3D-printed abutments to approach the performance of prefabricated abutments while offering customization advantages suggests a paradigm shift may be emerging in abutment selection criteria. From a clinical perspective, this study provides evidence that clinicians can consider 3D-printed abutments as a viable option in cases where both mechanical stability and a customized emergence profile are required—provided the specific manufacturing parameters and quality control are assured.

### Study limitations and future research

This study has several limitations: (1) Single implant connection type (internal hexagon) limits generalizability to other designs. (2) Load of 100 N is conservative compared to maximum occlusal forces; (3) Embedding and sectioning for SEM may introduce artifacts (minimized through slow-speed cutting under coolant). (4) The as-built condition of 3D-printed abutments, while providing standardization, may not fully represent clinical practice, as some laboratories perform additional finishing procedures. The absence of post-processing may represent a conservative estimate of clinical performance, as post-processing could potentially improve or, in some cases, compromise the fit and mechanical properties [18, 22]. (5) Sample size, while statistically powered, showed variability, particularly in customized abutment groups; the variability in customized groups (standard deviations up to 4.42 μm for implant-abutment gaps) may reflect inherent variations in the manufacturing processes, which could have clinical implications for individual restorations. (6) Surface roughness was discussed but not quantitatively measured.

Future studies should: (1) Validate findings across different implant systems (conical connections, external hex) under higher cyclic loads (150–300 N) to simulate long-term masticatory fatigue. (2) Investigate long-term biological implications of observed interfacial micro-gaps, particularly regarding microbial infiltration. (3) Conduct prospective randomized controlled clinical trials with adequate sample sizes and minimum 5-year follow-up periods to correlate in vitro mechanical advantages with long-term clinical outcomes, including implant survival, crestal bone loss, incidence of screw loosening, and prosthetic complications. (4) Include quantitative surface roughness measurements using profilometry or atomic force microscopy (AFM) on both external and internal surfaces. Surface roughness parameters should be correlated with torque retention and gap measurements to correlate topographical characteristics with mechanical performance and screw joint stability. (5) Evaluate effects of common clinical post-processing procedures on abutment performance.

## Conclusion

Within the limitations of this in vitro study, it can be concluded that:

1. Prefabricated abutments demonstrated the highest reverse torque values after cyclic loading, maintaining superior screw stability compared to both customized groups.
2. 3D-printed abutments showed significantly greater resistance to torque loss after cyclic loading compared to CAD-CAM-milled abutments, approaching the performance of prefabricated abutments.
3. The implant-abutment interface fit was comparable across groups, suggesting that for this connection design, the manufacturing technique had less influence on macroscopic fit than the connection geometry itself.
4. CAD-CAM-milled abutments exhibited the largest screw-abutment interface gaps and greatest torque loss, indicating potential limitations in their mechanical stability under cyclic loading conditions.

## Data Availability

Are available upon request from the corresponding author.
